# Adolescent cannabis use, depression and anxiety disorders in the Northern Finland Birth Cohort 1986

**DOI:** 10.1192/bjo.2021.967

**Published:** 2021-07-22

**Authors:** Antti Mustonen, Emily Hielscher, Jouko Miettunen, Alexander Denissoff, Anni-Emilia Alakokkare, James G. Scott, Solja Niemelä

**Affiliations:** Faculty of Medicine and Health Technology, Tampere University, Finland; Center for Life Course Health Research, University of Oulu, Finland; and Department of Psychiatry, Seinäjoki Central Hospital, Finland; QIMR Berghofer Medical Research Institute, Australia; School of Public Health, Faculty of Medicine, The University of Queensland, Australia; and Queensland Centre for Mental Health Research, Australia; Center for Life Course Health Research, University of Oulu, Finland; and Medical Research Center Oulu, Oulu University Hospital and University of Oulu, Finland; Department of Psychiatry, University of Turku, Finland; Center for Life Course Health Research, University of Oulu, Finland; and Department of Psychiatry, University of Turku, Finland; QIMR Berghofer Medical Research Institute, Australia; School of Public Health, Faculty of Medicine, The University of Queensland, Australia; Queensland Centre for Mental Health Research, Australia; and Metro North Mental Health Service, Australia; Department of Psychiatry, University of Turku, Finland; and Addiction Psychiatry Unit, Department of Psychiatry, Turku University Hospital, Finland

**Keywords:** Depressive disorders, anxiety disorders, epidemiology, cannabis, birth cohort

## Abstract

**Background:**

Cannabis use has been associated with increased risk of psychiatric disorders. However, associations between adolescent cannabis use, depression and anxiety disorders are inconsistently reported in longitudinal samples.

**Aims:**

To study associations of adolescent cannabis use with depression and anxiety disorders.

**Method:**

We used data from the Northern Finland Birth Cohort 1986, linked to nationwide registers, to study the association between adolescent cannabis use and depression and anxiety disorders until 33 years of age (until 2018).

**Results:**

We included 6325 participants (48.8% male) in the analyses; 352 (5.6%) participants reported cannabis use until 15–16 years of age. By the end of the follow-up, 583 (9.2%) participants were diagnosed with unipolar depression and 688 (10.9%) were diagnosed with anxiety disorder. Cannabis use in adolescence was associated with an increased risk of depression and anxiety disorders in crude models. After adjusting for parental psychiatric disorder, baseline emotional and behavioural problems, demographic factors and other substance use, using cannabis five or more times was associated with increased risk of anxiety disorders (hazard ratio 2.01, 95% CI 1.15–3.82), and using cannabis once (hazard ratio 1.93, 95% CI 1.30–2.87) or two to four times (hazard ratio 2.02, 95% CI 1.24–3.31) was associated with increased risk of depression.

**Conclusions:**

Cannabis use in adolescence was associated with an increased risk of future depression and anxiety disorders. Further research is needed to clarify if this is a causal association, which could then inform public health messages about the use of cannabis in adolescence.

Cannabis use has been associated with several mental health disorders in systematic reviews and meta-analyses, including psychotic disorders, where associations have been reported consistently.^1–4^ The current understanding of pathways from cannabis use to onset of these disorders is that of a complex and multifactorial process, with a range of contributing factors that span across an individual's life course.^5^

Among adolescents, there has been a trend in recent years to perceive that cannabis use has less risk of harm, including a lower risk perception of regular or daily cannabis use.^6,7^ By contrast, current evidence suggests that early onset and more frequent use is associated with a higher risk for cannabis-related adverse mental health and psychosocial outcomes, both in adolescence and later in life.^1,4,8^ Adolescence is a vulnerable neurodevelopmental period, where exposure to exogenous cannabinoids can interfere with neuronal maturation processes and have negative sequelae on adolescent brain development. Ultimately, the disruption of these maturation processes can influence the trajectories of mental health.^8,9^

## Previous studies on adolescent cannabis use, depression and anxiety disorders

Compared with the association with psychosis,^1,2^ the link between adolescent cannabis use and depression and anxiety disorders has been less studied in longitudinal prospective data-sets, and the studies have reported mixed findings.^4^ In general populations including both adults and adolescents, two meta-analyses have reported modest associations of cannabis use and depression,^3,10^ and two meta-analyses reported a slight increase in risk of anxiety.^11,12^ A recently published meta-analysis of prospective cohort studies^4^ focused on adolescent cannabis use, depression and anxiety until young adulthood, controlling for baseline symptoms. With a total sample size of 23 317, the meta-analysis reported a modest association for adolescent cannabis use and depression (odds ratio 1.37, 95% CI 1.16–1.62; seven studies) and a non-significant finding for anxiety disorders (odds ratio 1.18, 95% CI 0.84–1.67; three studies).^4^

Whether adolescent cannabis use is independently associated with depression and anxiety disorders remains unclear. In general, there are several other life-course factors that could confound the association of cannabis use and psychiatric disorders. Studies suggest that adolescent-onset substance use might emerge as self-medication for an underlying psychiatric disorder,^5,13^ or be influenced by early psychopathology.^14,15^ Furthermore, parental and familial factors may influence both offspring mental health and risk of substance use initiation.^16^ Research also suggests that use of cannabis is associated with increased risk of other substance use^17^ that may, in turn, increase the risk of psychiatric disorders.

Previous prospective studies on adolescent cannabis use, depression and anxiety disorders have typically derived depression and anxiety outcomes by using structured interviews or symptom scales.^17–25^ To our knowledge, there are no prospective birth cohort studies that have examined the association between adolescent cannabis use and risk of depression or anxiety disorders by linking questionnaire data to diagnoses based on data from registers. This is an alternative approach to study these phenomena in a setting where individuals have sought treatment for their mental illness. Furthermore, the authors of the recent meta-analysis emphasised the need for further prospective research about adolescent cannabis use, anxiety and depression.^4^

## Aims and objectives of this study

We utilised the Northern Finland Birth Cohort 1986 (*N* = 6325), which is linked to representative national registers including data on ICD-10 diagnoses. By using these data, we aimed to study longitudinal and temporal associations of adolescent cannabis use at 15–16 years of age, and subsequent depression and anxiety disorder until 33 years of age. We hypothesised that cannabis use in adolescence would be associated with depression and anxiety disorders until young adulthood, independent of parental psychiatric disorders, demographic factors, baseline emotional and behavioural problems and other substance use.

## Method

### Participants and data collection

**Fig. 1:**
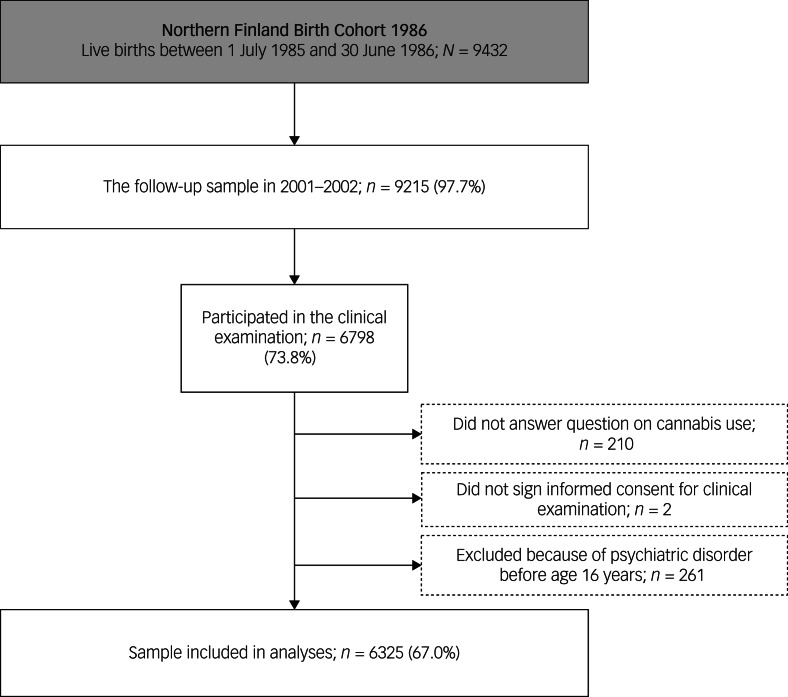
Flowchart of the current study from the Northern Finland Birth Cohort 1986.

The Northern Finland Birth Cohort 1986 is a general population-based birth cohort study including 99% of all births (*N* = 9432) with an expected date of birth between 1 July 1985 and 30 June 1986 from the two northernmost provinces in Finland.^26^ The mothers were recruited to this study from their first prenatal maternity clinic visit in 10th to 16th week of pregnancy, and the cohort participants have been followed up on ever since. A multidisciplinary follow-up study commenced in 2001–2002, when study members were aged 15–16 years. First, participants were sent self-report questionnaires (*n* = 9215) where they answered questions about their physical health, psychosocial well-being and smoking habits (*n* = 7344). Those who responded were invited to a clinical study. As a part of this collection the participants completed self-report questionnaires including questions on emotional and behavioural problems (Youth Self-Report [YSR])^27^ and substance use. The participants who signed the informed consent form and answered the question on cannabis use were included in the present study. To account for the effect of prior psychiatric disorders on study variables, we excluded participants with any prior psychiatric disorder before the age of 16 years. The final sample totalled 6325 (Fig. 1). The follow-up study in 2001–2002 was approved by the Ethics Committee of the Northern Ostrobothnia Hospital District in Finland (17 May 2006).

#### Measures

##### Exposure variable: cannabis use

The participants were asked ‘Have you ever used marihuana or hashish?’, with response options ‘never’, ‘once’, ‘two to four times’, ‘five or more times’ or ‘I use regularly’. For the main analysis, cannabis use was categorised into four groups, and two latter groups were pooled because of infrequent reporting. For inclusion of potential covariates, lifetime cannabis use was also studied as dichotomised (no/yes).

##### Outcome variables

Data on clinician-rated anxiety disorders (ICD-10 codes F40–44) and depression (ICD-10 codes F32.0–33.9, F34.1, F38.10)^28^ diagnoses were collected from 16 years of age until the end of 2018, when participants were aged 33 years. The onset age of each disorder was based on the first record of the diagnosis in registers. Diagnoses of psychiatric disorders were obtained from linkage to four nationwide registers providing substantial coverage on diagnosed psychiatric disorders in the wider community: The Register of Primary Health Care Visits 2011–2018 and the Care Register for Health Care 2001–2018 of the National Institute for Health and Welfare, the Medication Reimbursement Register of the Social Insurance Institution of Finland 2001–2005 and the Disability Pensions of the Finnish Centre for Pensions 2001–2016. The Care Register contains information on patients discharged from in-patient care, and since 1998, on specialised out-patient care. The Register of Primary Health Care Visits includes all out-patient primary healthcare delivered in Finland. For more information about the registers, see previous studies.^29–31^ The Finnish national registers are generally reliable,^32^ and typically provide data with minimal attrition from deaths and emigration compared with survey data.^33^

### Covariates

#### Emotional and behavioural problems in adolescence

Data on adolescent mental health were collected during the clinical study in 2001–2002, using the YSR.^27^ This measures symptoms of emotional or behavioural problems in adolescents aged 11–18 years. The participants were presented with 118 statements, including 29 items for externalising problems and 30 items for internalising problems. Responses were scored on a three-point scale, with the statements being not true (0), somewhat/sometimes true (1) or very true (2), based on how the participants had felt within the past 6 months. Scores for each item were summed to obtain a summary score for each subscale. If there were more than three answers missing in a subscale, the YSR subscale scores were excluded. If there were three or fewer missing values in a subscale, they were replaced by the mean value of items in that subscale for that person.^14^

#### Family structure, education level and parental psychiatric diagnosis

Information on structure of the family were collected by combining data from parents at birth and from the clinical study in 2001–2002. This data were categorised as ‘family with two parents’, where both parents lived together with the participant, and ‘other’, which consisted of all other families. Parental education level was collected from a postal questionnaire that was answered by parents in 2001–2002. Highest education level attained by a parent was divided into schooling for <12 years and schooling for ≥12 years (reference). Lifetime parental psychiatric diagnoses (ICD-10 codes F00–69, F80–99) were collected from the following nationwide registers: Register of Health Care during the years 1972–2018 (including in-patient care and visits to specialised out-patient healthcare since 1998), Disability Pensions of the Finnish Centre for Pensions (1965–2016) and The Register of Primary Health Care Visits (2011–2018).

#### Cigarette smoking, alcohol use and illicit substance use

Data on lifetime illicit substance use and frequent alcohol intoxications were collected with a questionnaire that the participants received during the clinical study in 2001–2002. The participants were asked ‘Have you used ecstasy, heroin, cocaine, amphetamine, LSD or other similar intoxicating drugs?’ The participants were categorised into the ‘yes’ group if they reported lifetime use of these substances. Frequent alcohol intoxications were questioned as ‘Have you been drunk during the past year? (0, 1–2, 3–5, 6–9, 10–19, 20–39 or ≥40 times)’, and this was categorised as ‘Have you been drunk during the past year? ten times or more (no/yes)’. Data on regular cigarette smoking was collected from the postal questionnaire. The participants were asked if they currently smoked cigarettes daily (one or more cigarettes per day), with options no/yes.

### Statistical methods

We used *χ*^2^-test or Fisher's exact test to assess the relationship of lifetime cannabis use, depression, anxiety disorders and covariates. Those reaching statistical significance *P* < 0.10 for all of the outcomes were accepted to final multivariable models. Linear regression and multicollinearity diagnostics with variance inflation factor (VIF) scores were used to examine possible correlation between multiple covariates (fully adjusted model 3). VIF > 5 was considered as an indicator of multicollinearity.

Cox regression analysis with hazard ratios and 95% confidence intervals was used to examine the association of adolescent cannabis use with onset of depression and anxiety disorders. The participants diagnosed with any psychiatric disorder at baseline were excluded (*n* = 261). Dates of emigration (*n* = 256) and death (*n* = 51) were used as censoring points in survival analyses. Among these, there were participants that emigrated but moved back to Finland and were later diagnosed with anxiety disorder (*n* = 19) or depression (*n* = 12). They were censored at emigration date in survival analyses because register data for diagnoses were not available for the emigration period. Two of the censored cases were excluded in survival analyses because of death or emigration before the earliest possible event in stratum, i.e., they participated in clinical examination but emigrated or died before onset of first depression or anxiety disorder case in the data. Cox proportional hazard assumption was studied with hazard logarithms and scaled Schoenfeld residuals.

To consider the effect of potential confounders to depression and anxiety disorder outcomes, the following models were used. We studied the models separately by YSR internalising and YSR externalising symptom scores to examine whether these associations varied by baseline symptomology. In model 1, we used (a) YSR internalising symptom scores and (b) YSR externalising symptom scores, and these covariates are presented as model Xa or model Xb in subsequent models; model 2 further included daily smoking, frequent alcohol intoxications in the past year and illicit substance use other than cannabis at baseline; model 3 further included parental psychiatric disorder and family structure. Survival curves were computed for Model 3b separately for depression and anxiety disorders.

We also examined a dose–response effect between cannabis use and depression and anxiety disorders, using a categorical cannabis use variable (never, once, two to four times, five or more times) as continuous in Cox regression analyses with hazard ratios and 95% confidence intervals.

Sensitivity analysis, E-values, attrition analysis and missing data are described in the Supplementary material available at https://doi.org/10.1192/bjo.2021.967.

Statistical analyses were conducted with SPSS statistical software for Windows (IBM SPSS Statistics, version 25) except for E-values and examination of Cox proportional hazard assumption, which were analysed with R for Windows (R Foundation for Statistical Computing, version 3.6.0; R Core Team, Vienna, Austria) packages survival, survminer and Epi.

## Results

The sample totalled 6325 participants, of which 352 adolescents (5.6%) reported any cannabis use until 15–16 years of age. Among those who emigrated during the follow-up and were excluded from the study, 16 out of 256 (6.3%) reported any cannabis use. During the follow-up (from ages 16 to 33 years), 583 (9.2% of the total sample) were diagnosed with any depression and 688 (10.9% of the total sample) were diagnosed with any anxiety disorder. For the sample demographics, please see Table 1. No significant collinearity was seen between variables (all VIFs < 1.3).

**Table 1:** Association between covariates and cannabis use, depression and anxiety disorders in Northern Finland Birth Cohort 1986

				Cannabis use frequency					Anxiety disorder					Depression				
	Total, *n*	*N* = 6325		No cannabis use, *n* = 5973		Once or more, *n* = 352		*P*-value	No anxiety disorder, *n* = 5637		Anxiety disorder, *n* = 688		*P*-value	No depression, *n* = 5742		Depression, *n* = 583		*P*-value
		*n*	%	*n*	%	*n*	%		*n*	%	*n*	%		*n*	%	*n*	%	
Gender	6325																	
Male		3089	48.8	2929	49.0	160	45.5	0.191	2847	50.5	242	35.2	<0.001	2888	50.3	201	34.5	<0.001
Female		3236	51.2	3044	51.0	192	54.5		2790	49.5	446	64.8		2854	49.7	382	65.5	
Family structure	5413																	
Other		1102	20.4	1004	19.6	98	33.4	<0.001	945	19.5	157	27.3	<0.001	960	19.5	142	28.7	<0.001
Family with two parents		4311	79.6	4116	80.4	195	66.6		3893	80.5	418	72.7		3958	80.5	353	71.3	
Parental education	5066																	
<12 years		3050	60.2	2905	60.5	145	54.9	0.072	2723	59.9	327	62.8	0.208	2783	60.3	267	59.7	0.830
≥12 years		2016	39.8	1897	39.5	119	45.1		1822	40.1	194	37.2		1836	39.7	180	40.3	
Cannabis use	6325																	
No		5973	94.4	352	100.0	0	0.0	-	5352	94.9	621	90.3	<0.001	5461	95.1	512	87.8	<0.001
Yes		352	5.6	0	0.0	5973	100.0		285	5.1	67	9.7		281	4.9	71	12.2	
Daily smoking	5871																	
No		5151	87.7	4977	89.7	174	54.2	<0.001	4624	88.5	527	81.8	<0.001	4712	88.5	439	80.3	<0.001
Yes		720	12.3	573	10.3	147	45.8		603	11.5	117	18.2		612	11.5	108	19.7	
Other illicit drug use	6299																	
No		6268	99.5	5942	99.9	326	92.9	<0.001^a^	5593	99.6	675	98.4	<0.001^a^	5696	99.6	572	98.6	0.006^a^
Yes		31	0.5	6	0.1	25	7.1		20	0.4	11	1.6		23	0.4	8	1.4	
Alcohol intoxication ≥10 times in the past year	6167																	
No		5024	81.5	4910	84.4	114	32.9	<0.001	4510	82.1	514	76.3	<0.001	4598	82.2	426	74.2	<0.001
Yes		1143	18.5	910	15.6	233	67.1		983	17.9	160	23.7		995	17.8	148	25.8	
Parental psychiatric disorder	6325																	
No		4046	64.0	3836	64.2	210	59.7	0.083	3693	65.5	353	51.3	<0.001	3742	65.2	304	52.1	<0.001
Yes		2279	36.0	2137	35.8	142	40.3		1944	34.5	335	48.7		2000	34.8	279	47.9	
Youth Self-Report		Mean	s.d.	Mean	s.d.	Mean	s.d.		Mean	s.d.	Mean	s.d.		Mean	s.d.	Mean	s.d.	
Internalising symptoms	5876	9.6	7.5	9.4	7.4	11.8	8.9		9.3	7.3	12.0	8.6		9.2	7.2	13.4	8.9	
Externalising symptoms	5892	13.8	7.9	13.5	7.6	20.3	9.6		13.6	7.7	16.2	8.9		13.5	7.7	16.9	8.8	

a. Fisher's exact test.

**Fig. 2:**
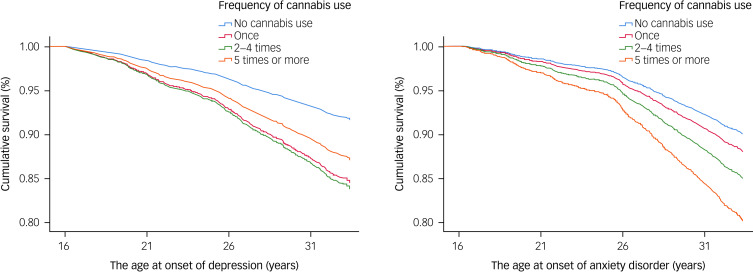
Survival curves for the association between adolescent cannabis use, depression and anxiety disorders.

The models were consistent with the Cox proportional hazard assumption, and time dependence was not observed. In crude models, using cannabis once or more frequently in adolescence was associated with increased risk of subsequent depression and anxiety disorder during the follow-up period (see Table 2). After adjustment for YSR internalising symptoms in adolescence (model 1a), these associations attenuated but remained statistically significant. After adjustment for YSR externalising symptoms in adolescence (model 1b), the associations were statistically significant for cannabis use five or more times and anxiety disorders, and cannabis use once or two to four times and depression. The associations attenuated further after adjustments for daily smoking, frequent alcohol intoxications in the past year, illicit substance use other than cannabis, parental psychiatric disorder, and family structure. In fully adjusted models 3a and 3b, cannabis use five or more times in adolescence was associated with an increased risk of subsequent anxiety disorder (hazard ratio 2.01–2.20), and cannabis use once or two to four times was associated with an increased risk of subsequent depression (cannabis use once: hazard ratio 1.93–2.01; cannabis use two to four times: hazard ratio 2.02). For all of the results and confidence intervals, see Table 2. The survival curves for model 3b are presented for both depression and anxiety disorders in Figure 2.

**Table 2:** Association between cannabis use, depression and anxiety disorders in Northern Finland Birth Cohort 1986

			No cannabis use	Once			2–4 times			≥5 times		
Frequency of cannabis use		Sample size	Cases	Cases	Hazard ratio	95% CI	Cases	Hazard ratio	95% CI	Cases	Hazard ratio	95% CI
Depression	Crude	6323	500	38	**2.66**	**1.91–3.70**	21	**2.51**	**1.62–3.88**	12	**2.56**	**1.45–4.54**
	Model 1a	5874	465	36	**2.40**	**1.70–3.37**	20	**2.26**	**1.44–3.53**	11	**2.14**	**1.17–3.90**
	Model 2a	5646	452	36	**2.06**	**1.43–2.95**	20	**1.88**	**1.18–3.01**	11	1.42	0.71–2.84
	Model 3a	5021	395	30	**2.01**	**1.36–2.98**	18	**2.02**	**1.23–3.31**	11	1.64	0.82–3.29
	Model 1b	5890	468	36	**2.11**	**1.49–2.98**	20	**2.02**	**1.29–3.18**	11	1.73	0.94–3.18
	Model 2b	5663	456	36	**1.97**	**1.37–2.83**	20	**1.88**	**1.18–3.00**	11	1.34	0.68–2.62
	Model 3b	5038	397	30	**1.93**	**1.30–2.87**	18	**2.02**	**1.24–3.31**	11	1.58	0.80–3.11
Anxiety disorder	Crude	6323	603	29	**1.62**	**1.11–2.35**	19	**1.88**	**1.19–2.97**	18	**3.36**	**2.10–5.37**
	Model 1a	5874	561	28	**1.56**	**1.06–2.28**	18	**1.77**	**1.11–2.84**	16	**2.98**	**1.81–4.91**
	Model 2a	5646	543	28	1.36	0.91–2.02	17	1.46	0.88–2.40	16	**1.94**	**1.07–3.53**
	Model 3a	5021	465	22	1.31	0.84–2.04	15	1.63	0.95–2.78	15	**2.20**	**1.18–4.08**
	Model 1b	5890	566	28	1.37	0.93–2.02	18	1.57	0.98–2.52	16	**2.53**	**1.53–4.18**
	Model 2b	5663	548	28	1.27	0.85–1.90	17	1.40	0.85–2.31	16	**1.81**	**1.02–3.23**
	Model 3b	5038	468	22	1.21	0.77–1.90	15	1.55	0.91–2.64	15	**2.01**	**1.15–3.82**

Statistically significant results are shown in bold. Model 1a: Youth Self-Report internalising symptoms; model 2a: Youth Self-Report internalising symptoms, daily smoking, other illicit substance use, frequent alcohol intoxications in the past year; model 3a: Youth Self-Report internalising symptoms, daily smoking, other illicit substance use, frequent alcohol intoxications in the past year, family structure, parental psychiatric disorder; model 1b: Youth Self-Report externalising symptoms; model 2b: Youth Self-Report externalising symptoms, daily smoking, other illicit substance use, frequent alcohol intoxications in the past year; model 3b: Youth Self-Report externalising symptoms, daily smoking, other illicit substance use, frequent alcohol intoxications in the past year, family structure, parental psychiatric disorder.

Using model 3a, cannabis use was associated with anxiety disorder and depression in a dose–response manner for anxiety disorder (hazard ratio 1.32, 95% CI 1.09–1.59) and depression (hazard ratio 1.39, 95% CI 1.14–1.67).

The results from primary analyses were stable with our sensitivity analysis (Supplementary Table 2), as the hazard ratios were generally similar in models with or without excluding those with the onset of any psychiatric disorder before 16 years of age. However, using cannabis two to four times remained weakly associated with anxiety disorders in the sample that was not restricted for prior psychiatric disorders (see Supplementary Table 2). Furthermore, our sensitivity analysis using E – values suggested the statistically significant findings were relatively robust to unmeasured confounding, as point estimates for E-values were 3.44–3.82 (model 3a) for these associations of adolescent cannabis use, depression and anxiety disorders (see Supplementary Table 3).

## Discussion

Using a prospective birth cohort sample, we report modest associations of adolescent cannabis use and increased risk of future depression and anxiety disorder. There were statistically significant associations between adolescent cannabis use and depression and anxiety disorders after adjusting for baseline externalising and internalising symptoms. This suggests that cannabis use may increase the risk of depression and anxiety disorders independently of adolescent psychopathology. Moreover, these associations were independent of other substance use and familial factors. Furthermore, our trend analysis suggested a weak dose–response effect. However, the risk of depression associated with cannabis use increased inconsistently, suggesting a lack of power for this analysis.

The recent meta-analysis by Gobbi et al^4^ reported a modest association between cannabis use and depression (odds ratio 1.37; two out of seven studies reported a statistically significant association) and a non-significant association for anxiety disorders (odds ratio 1.18; zero out of three studies reported a statistically significant association). In contrast to the meta-analysis by Gobbi et al,^4^ we report an association between frequent cannabis use and subsequent anxiety disorders by using prospective birth cohort linked national register data. Possible reasons for these findings being inconsistent with previous studies include smaller sample sizes in studies included in the meta-analysis (*n* = 1234–1943 *v*. *n* = 5038 here), differing follow-up times and different study designs.^18–20^ The examination of adolescent cannabis use and depression produced statistically significant findings, as did the meta-analysis by Gobbi et al.^4^ However, our results were influenced by infrequent reporting of cannabis use five or more times introducing power issues. Considering previous meta-analyses have reported dose-dependent associations between cannabis use and depression in the general population,^3,10^ the risk for depression should plausibly increase with the quantity and/or frequency of cannabis use.

Adolescent-onset substance use might occur as self-medication for underlying psychiatric disorder.^5,13^ The studies that were included in meta-analysis by Gobbi et al^4^ all controlled for baseline symptoms, thus providing data on temporality of the association between cannabis use, depression and anxiety disorders. In this study, we adjusted for YSR externalising and internalising symptoms, to reflect baseline psychopathology. In general, adjusting for externalising symptoms attenuated the hazard ratio more than internalising symptoms, which is plausible as substance use is generally more influenced by externalising behaviours.^14,34^ However, the association of adolescent cannabis use, depression and anxiety disorders remained statistically significant after adjusting for YSR scores in our analysis. This suggests a temporal association between adolescent cannabis use, depression and anxiety disorders, and reverse causation does not entirely explain these findings. This is further supported by the fact that we excluded participants who were diagnosed with psychiatric disorders at baseline.

Previous literature suggests that family structure and parental psychiatric disorder may influence offspring mental health and substance use involvement via genetic and environmental factors.^16^ In addition, adolescent cannabis use has been associated with increased risk of other substance use,^17^ which could increase the risk of depression or anxiety disorders.^14^ Thus, these could confound the postulated associations of cannabis use and psychiatric disorders. However, after further adjusting for substance use other than cannabis, family structure and parental psychiatric disorder, cannabis use remained statistically significantly associated with future anxiety disorder and depression. This suggests that adolescent cannabis use may be an independent contributing factor in the trajectory of these disorders. However, the findings concerning depression were not consistent with a dose–response relationship, and we emphasise the need for studies in other samples.

There are several strengths to this study, including the sample size, minimal loss to death or emigration, and high genetic and ethnic homogeneity allowing for robust examination of the associations between variables of interest (cannabis use, anxiety, depression) and potential confounding factors.

However, there are also several limitations. Males and individuals from urban areas were less likely to participate in the follow-up study in 2001–2002. This could have introduced selection bias. Data on lifetime cannabis use at 15–16 years of age were collected by self-report, potentially resulting in underreporting. There were few participants that reported using cannabis five or more times, which introduced power issues. Furthermore, information concerning use of cannabis and other substances was available for only one time point. Thus, we did not have data on whether cannabis use of the participants continued. Furthermore, the categorisation of our cannabis use variable (never, once, two to four times, five or more times) does not reflect the important aspects of regular/daily use. This is likely to lead to underestimation of the association between cannabis use, depression and anxiety disorders. Also, we were unable to specify what kind of cannabis products the participants had used, including the tetrahydrocannabinol potency, which has markedly increased in past years.^35^ Although register diagnoses for cannabis use disorder were available, we could not reliably incorporate this data to our analysis because of the low cumulative incidence of this outcome (0.32%). The YSR data reflects the participants’ situation at 15–16 years of age, and thus it is possible that there were adolescents who experienced depressive and anxiety symptoms earlier than when these data were collected, before the onset of substance use, which may have led to reverse causality. Finally, although E-values suggested that the results were moderately robust to unmeasured confounding, we were not able to consider the influences of genetics and childhood adversity.

In conclusion, cannabis use during adolescence was associated with a modest increase in risk of subsequent depression and anxiety disorders. More prospective studies with large sample sizes, multiple time points of substance use assessment, and adequate assessment for potential confounders (e.g. childhood trauma) and moderators (e.g. ongoing cannabis use) are needed to address the life-course association between cannabis use and anxiety disorders and depression.

## Data Availability

Northern Finland Birth Cohort 1986 data is available from the University of Oulu, Infrastructure for Population Studies. Permission to use the data can be applied for research purposes via electronic material request portal. In the use of data, we follow the European Union General Data Protection Regulation (679/2016) and Finnish Data Protection Act. The use of personal data is based on cohort participant's written informed consent at their latest follow-up study, which may cause limitations to its use. Please contact the Northern Finland Birth Cohort 1986 project centre (NFBCprojectcenter@oulu.fi) and visit the cohort website (www.oulu.fi/nfbc) for more information.

## Supplementary material

For supplementary material accompanying this paper visit http://dx.doi.org/10.1192/bjo.2021.967. click here to view supplementary material
