# Supplementary material for: Adolescent cannabis use, depression and anxiety disorders in the Northern Finland Birth Cohort 1986
Source: BJPsych Open. 2021 Jul 22;7(4):e137. doi: 10.1192/bjo.2021.967 (PMC8329769; doi:10.1192/bjo.2021.967)
Supplement: Supplementary file 1 [file S2056472421009674sup001.docx]

**Sensitivity analysis, E – values, attrition analysis and missing data.**

**Table 1. Frequencies of covariates within different cannabis use categories in crude and fully adjusted models.**

**Table 2. Sensitivity analysis for the association between cannabis use, depression, and anxiety disorders in the Northern Finland Birth Cohort 1986.**

**Table 3. E-values for the association between cannabis use, depression, and anxiety disorders in the Northern Finland Birth Cohort 1986.**

**Table 4. Sensitivity analysis for the association between cannabis use, depression, and anxiety disorders in the Northern Finland Birth Cohort 1986.**

**Sensitivity analysis, E – values, attrition analysis and missing data**

Previous attrition analyses of this NFBC1986 sample have shown that fewer males (64% v. 71%; p<0.001), individuals living in urban areas (66% v. 71%, p<0.001) and individuals with parental psychiatric disorder (58% v. 69%, p<0.001) participated in the 15-16 year follow up study (1). Participants with missing data in questionnaires were excluded if the items used as covariates were not reported. This data is reported in Table 1 in crude vs. fully adjusted model.

To assess the stability of our results, a sensitivity analysis was performed. We used Cox-regression analysis with hazard ratios (HR) and 95%CI in Model 3a without restricting the sample for participants psychiatric disorder prior age 16 years (Table 2). As follow-up periods of cohort participants and their parents coincide, another sensitivity analysis was conducted. This analysis considered participants having parental psychiatric disorder if this diagnosis were made prior to the cohort study period i.e., before the age 16 years (Table 4).

Furthermore, we calculated E – values to study whether our findings could be explained by possible unmeasured confounding (Table 3). E – value is an alternative approach for sensitivity analysis in observational studies where E-values with lower bound of the CI indicate the minimum strength of unmeasured confounders´ association with independent (here cannabis use) and dependent variable (here depression and anxiety disorders) that could explain away the observed association between exposure and outcome. Small E-values (lowest possible 1.0) suggest that relatively weak unmeasured confounding would be required to affect the observed association, while larger E-values suggest that observed associations are robust to unmeasured confounding (2,3).

**References:**

1. Miettunen J, Murray GK, Jones PB, Mäki P, Ebeling H, Taanila A, et al. Longitudinal associations between childhood and adulthood externalizing and internalizing psychopathology and adolescent substance use. Psychol Med 2014;44(8):1727–38.

2. Van Der Weele TJ, Ding P. Sensitivity analysis in observational research: Introducing the E-Value. Ann Intern Med. 2017;167(4):268–74.

3. Haneuse S, Vanderweele TJ, Arterburn D. Using the E-Value to Assess the Potential Effect of Unmeasured Confounding in Observational Studies. JAMA 2019; 321:602–3.

| **Table 1. Frequencies of covariates within different cannabis use categories in crude and fully adjusted models.** | | | | | | | | | | |
| --- | --- | --- | --- | --- | --- | --- | --- | --- | --- | --- |
|  |  | Crude model | |  |  |  | Model 3a | |  |  |
|  | No cannabis use | Once | 2-4 times | 5 times of more |  | No cannabis use | Once | 2-4 times | 5 times of more |  |
| **Family structure** |  |  |  |  |  |  |  |  |  |  |
| Other | 1004 | 46 | 26 | 26 |  | 935 | 41 | 26 | 25 |  |
|  | *19,6%* | *30,1%* | *29,5%* | *50,0%* |  | *19,7%* | *28,9%* | *32,1%* | *51,0%* |  |
| Family with two parents | 4116 | 107 | 62 | 26 |  | 3814 | 101 | 55 | 24 |  |
|  | *80,4%* | *69,9%* | *70,5%* | *50,0%* |  | *80,3%* | *71,1%* | *67,9%* | *49,0%* |  |
| **Daily smoking** |  |  |  |  |  |  |  |  |  |  |
| No | 4977 | 95 | 56 | 23 |  | 4307 | 83 | 45 | 19 |  |
|  | *89,7%* | *56,9%* | *56,6%* | *41,8%* |  | *90,7%* | *58,5%* | *55,6%* | *38,8%* |  |
| Yes | 573 | 72 | 43 | 32 |  | 442 | 59 | 36 | 30 |  |
|  | *10,3%* | *43,1%* | *43,4%* | *58,2%* |  | *9,3%* | *41,5%* | *44,4%* | *61,2%* |  |
| **Other illicit drug use** |  |  |  |  |  |  |  |  |  |  |
| No | 5942 | 175 | 105 | 46 |  | 4744 | 137 | 79 | 38 |  |
|  | *99,9%* | *96,2%* | *97,2%* | *75,4%* |  | *99,9%* | *96,5%* | *97,5%* | *77,6%* |  |
| Yes | 6 | 7 | 3 | 15 |  | 5 | 5 | 2 | 11 |  |
|  | *0,1%* | *3,8%* | *2,8%* | *24,6%* |  | *0,1%* | *3,5%* | *2,5%* | *22,4%* |  |
| **Alcohol intoxication 10** ≥ **times past year** |  |  |  |  |  |  |  |  |  |  |
| No | 4910 | 71 | 25 | 18 |  | 4028 | 56 | 21 | 16 |  |
|  | *84,4%* | *39,2%* | *23,8%* | *29,5%* |  | *84,8%* | *39,4%* | *25,9%* | *32,7%* |  |
| Yes | 910 | 110 | 80 | 43 |  | 721 | 86 | 60 | 33 |  |
|  | *15,6%* | *60,8%* | *76,2%* | *70,5%* |  | *15,2%* | *60,6%* | *74,1%* | *67,3%* |  |
| **Parental psychiatric disorder** |  |  |  |  |  |  |  |  |  |  |
| No | 3836 | 114 | 64 | 32 |  | 3071 | 90 | 49 | 26 |  |
|  | *64,2%* | *62,3%* | *59,3%* | *52,5%* |  | *64,7%* | *63,4%* | *60,5%* | *53,1%* |  |
| Yes | 2137 | 69 | 44 | 29 |  | 1678 | 52 | 32 | 23 |  |
|  | *35,8%* | *37,7%* | *40,7%* | *47,5%* |  | *35,3%* | *36,6%* | *39,5%* | *46,9%* |  |
| **Anxiety disorder** |  |  |  |  |  |  |  |  |  |  |
| No | 5352 | 154 | 88 | 43 |  | 4268 | 120 | 65 | 34 |  |
|  | *89,6%* | *84,2%* | *81,5%* | *70,5%* |  | *89,9%* | *84,5%* | *80,2%* | *69,4%* |  |
| Yes | 621 | 29 | 20 | 18 |  | 481 | 22 | 16 | 15 |  |
|  | *10,4%* | *15,8%* | *18,5%* | *29,5%* |  | *10,1%* | *15,5%* | *19,8%* | *30,6%* |  |
| **Depression** |  |  |  |  |  |  |  |  |  |  |
| No | 5461 | 145 | 87 | 49 |  | 4344 | 112 | 63 | 38 |  |
|  | *91,4%* | *79,2%* | *80,6%* | *80,3%* |  | *91,5%* | *78,9%* | *77,8%* | *77,6%* |  |
| Yes | 512 | 38 | 21 | 12 |  | 405 | 30 | 18 | 11 |  |
|  | *8,6%* | *20,8%* | *19,4%* | *19,7%* |  | *8,5%* | *21,1%* | *22,2%* | *22,4%* |  |

Model 3a: YSR Int, daily smoking, other illicit substance use, alcohol intoxication 10 ≥ times past year, family structure, parental psychiatric disorder.

Model 3a: YSR Int, daily smoking, other illicit substance use, alcohol intoxication 10 ≥ times past year, family structure, parental psychiatric disorder.

| **Table 2.** **Sensitivity analysis for the association between cannabis use, depression, and anxiety disorders in the Northern Finland Birth Cohort 1986.** | | | | | | | | | | | | | | | | |
| --- | --- | --- | --- | --- | --- | --- | --- | --- | --- | --- | --- | --- | --- | --- | --- | --- |
|  |  |  |  |  |  |  | | |  |  | | |  |  |  |  |
| **Frequency of cannabis use** |  |  |  | **No cannabis use** |  | **Once** | | |  | **2-4 times** | | |  | **5 times or more** | | |
|  | |  | Sample size | Cases |  | Cases | HR | 95% CI |  | Cases | HR | 95% CI |  | Cases | HR | 95% CI |
| **Depression** | | Model 3a | 5200 | 442 |  | 35 | **2.05** | **1.42-2.95** |  | 24 | **2.30** | **1.49-3.55** |  | 13 | 1.79 | 0.96-3.34 |
| **Anxiety disorder** | | Model  3a | 5200 | 509 |  | 23 | 1.19 | 0.77-1.83 |  | 19 | **1.70** | **1.05-2.76** |  | 17 | **2.36** | **1.34-4.16** |

| **Table 3. E-values for the association between cannabis use, depression, and anxiety disorders in the Northern Finland Birth Cohort 1986.** | | | | | | | | | | | | | | | | | | | | | | | | |
| --- | --- | --- | --- | --- | --- | --- | --- | --- | --- | --- | --- | --- | --- | --- | --- | --- | --- | --- | --- | --- | --- | --- | --- | --- |
|  |  |  |  | |  | |  | | | |  | |  | | | |  | |  | |  |  | |  |
| **Frequency of cannabis use** |  |  |  | **Once** | | | | |  | **2-4 times** | | | | |  | **5 times or more** | | | | | | |  |  |
|  | |  |  |  | | HR | | Lower bound of 95% CI |  |  | | HR | | Lower bound of 95% CI |  |  | | HR | | Lower bound of 95% CI | | |  |  |
| **Depression** | | Crude |  |  | | 4.76 | | 3.24 |  |  | | 4.45 | | 2.62 |  |  | | 4.56 | | 2.25 | | |  |  |
|  | | Model 1a |  |  | | 4.23 | | 2.81 |  |  | | 3.94 | | 2.24 |  |  | | 3.70 | | 1.63 | | |  |  |
|  | | Model 2a |  |  | | 3.53 | | 2.22 |  |  | | 3.17 | | 1.64 |  |  | | 2.19 | | 1 | | |  |  |
|  | | Model 3a |  |  | | 3.44 | | 2.06 |  |  | | 3.45 | | 1.76 |  |  | | 2.66 | | 1 | | |  |  |
| **Anxiety disorder** | | Crude |  |  | | 2.61 | | 1.47 |  |  | | 3.17 | | 1.67 |  |  | | 6.17 | | 3.62 | | |  |  |
|  | | Model 1a |  |  | | 2.49 | | 1.32 |  |  | | 2.95 | | 1.46 |  |  | | 5.42 | | 3.03 | | |  |  |
|  | | Model 2a |  |  | | 2.05 | | 1 |  |  | | 2.28 | | 1 |  |  | | 3.30 | | 1.34 | | |  |  |
|  | | Model  3a |  |  | | 1.94 | | 1 |  |  | | 2.64 | | 1 |  |  | | 3.82 | | 1.65 | | |  |  |

Model 3a: YSR Int, daily smoking, other illicit substance use, alcohol intoxication 10 ≥ times past year; family structure, parental psychiatric disorder.

| **Table 4. Sensitivity analysis for the association between cannabis use, depression, and anxiety disorders in the Northern Finland Birth Cohort 1986.** | | | | | | | | | | | | | | | | |
| --- | --- | --- | --- | --- | --- | --- | --- | --- | --- | --- | --- | --- | --- | --- | --- | --- |
|  |  |  |  |  |  |  | | |  |  | | |  |  |  |  |
| **Frequency of cannabis use** |  |  |  | **No cannabis use** |  | **Once** | | |  | **2-4 times** | | |  | **5 times or more** | | |
|  | |  | Sample size | Cases |  | Cases | HR | 95% CI |  | Cases | HR | 95% CI |  | Cases | HR | 95% CI |
| **Depression** | | Model 3a | 5021 | 395 |  | 30 | **1.95** | **1.32-2.88** |  | 18 | **2.04** | **1.24-3.35** |  | 11 | 1.62 | 0.80-3.31 |
| **Anxiety disorder** | | Model  3a | 5021 | 465 |  | 22 | 1.27 | 0.81-1.99 |  | 15 | 1.61 | 0.95-2.75 |  | 15 | **2.11** | **1.13-3.95** |

Model 3a: YSR Int, daily smoking, other illicit substance use, alcohol intoxication 10 ≥ times past year, family structure, parental psychiatric disorder.
